# Pulmonary Artery Banding for Dilated Cardiomyopathy in Children: Returning to the Bench from Bedside

**DOI:** 10.3390/children9091392

**Published:** 2022-09-14

**Authors:** Matteo Ponzoni, Biagio Castaldi, Massimo A. Padalino

**Affiliations:** 1 Pediatric and Congenital Cardiac Surgery Unit, Department of Cardiac, Thoracic, Vascular Sciences and Public Health, University of Padua, 35122 Padua, Italy; 2 Pediatric Cardiology Unit, Department of Woman’s and Child’s Health, University of Padua, 35122 Padua, Italy

**Keywords:** pulmonary artery banding, dilated cardiomyopathy, review

## Abstract

Current treatment paradigms for end-stage dilated cardiomyopathy (DCM) in children include heart transplantation and mechanical support devices. However, waitlist mortality, shortage of smaller donors, time-limited durability of grafts, and thrombo-hemorrhagic events affect long-term outcomes. Moreover, both these options are noncurative and cannot preserve the native heart function. Pulmonary artery banding (PAB) has been reinvented as a possible “regenerative surgery” to retrain the decompensated left ventricle in children with DCM. The rationale is to promote positive ventricular–ventricular interactions that result in recovery of left ventricular function in one out of two children, allowing transplantation delisting. Although promising, global experience with this technique is still limited, and several surgical centers are reluctant to adopt PAB since its exact biological bases remain unknown. In the present review, we summarize the clinical, functional, and molecular known and supposed working mechanisms of PAB in children with DCM. From its proven efficacy in the clinical setting, we described the macroscopic geometrical and functional changes in biventricular performance promoted by PAB. We finally speculated on the possible underlying molecular pathways recruited by PAB. An evidence-based explanation of the working mechanisms of PAB is still awaited to support wider adoption of this surgical option for pediatric heart failure.

## 1. Introduction

Pediatric heart failure (HF) represents an important cause of morbidity and mortality in childhood, with various causes (congenital, genetic, or acquired early in utero or after birth). Management of end-stage HF in infants and children remains challenging. The ultimate therapy for end-stage HF is heart transplantation (HTX). However, the survival rates after HTX are suboptimal since the patient’s prognosis drastically decreases with time, with the overall 25-year survival as low as 37% [1]. This makes pediatric HTX a not-so-appealing option, especially for infants or smaller children affected by end-stage HF, whose expectancy of life, given currently available treatment options, is drastically limited with respect to healthy peers.

In the present review, we collate the current knowledge on the use of pulmonary artery banding (PAB) as an alternative treatment option for left ventricular (LV) rehabilitation in children affected by dilated cardiomyopathy (DCM), addressing its observed and speculated biological working mechanisms. Starting from our clinical experience, we propose novel suggestions and refinements of current selection criteria, which we learnt from our cohort of children with DCM treated with PAB, for a more cautious application of this technique. Moreover, we present our hypotheses on the possible underlying molecular pathways recruited by this technique, with the aim of stimulating targeted experimental research on the regenerative properties of PAB. A wider scientific consensus on the efficacy of PAB in reverting HF is still awaited to support the adoption of PAB as part of treatment paradigms for pediatric HF.

## 2. Background/The State of the Art

In the pediatric population, HF is still a field where more questions than answers exist. The incidence of HF in European countries ranges from 1 to 3 cases per 100,000 children, and the main etiologies are congenital heart diseases and cardiomyopathies [2]. Dilated cardiomyopathy in children has a wide spectrum of causes, but in most of the cases, a leading factor cannot be detected, limiting specific treatments. Prognosis remains poor, with more than 40% of children expiring or requiring HTX, especially in more compromised functional classes and when a marked reduction in LV contractile function is present [3,4].

When medical therapy is ineffective, patients are evaluated for HTX and/or mechanical circulatory support (MCS). Unfortunately, the number of listed children still exceeds the number of potential donors, and waitlist mortality can reach 30% [1,5]. Moreover, chronic rejection limits the long-term outcomes of patients, whose median survival is <20 years [1]. This means that more than 50% of children with end-stage HF will not reach adulthood.

A growing number of pediatric patients are nowadays bridged to HTX with MCS [6,7]. Guaranteeing adequate organ perfusion and LV unloading, the multiorgan function is optimized, and post-transplantation outcomes improve [7,8].

However, both these strategies are noncurative and turn the primary cardiac pathology into a chronic systemic disease. The immunosuppressive regimen and related risks of infection and malignancy represent the main concerns in pediatric HTX [9,10], as well as the hemorrhagic and thromboembolic morbidities in MCS [6,8,11]. Moreover, smaller patients can benefit from exclusively extracorporeal MCS devices (i.e., the Berlin Heart), which negatively affect the length of hospitalization, social costs, and quality of life [11]. Less than 10% of patients are weaned from MCS [6], and HF recurrence is frequent [12], underlying the absence of a complete myocardial recovery. In a very recent multicenter review of 193 children and adolescents implanted with a ventricular assist device, only 25 patients (13%) were weaned from MCS [13]. In this paper, the multivariate analysis proved that younger age, shorter duration of support, and an acute manifestation of HF (i.e., myocarditis) were predictors of successful weaning from the ventricular assist device. In this light, the usually chronic clinical course of DCM, together with the profound tissue-level alterations and fibrotic changes that are observed in affected hearts, may imply even fewer chances of sustained myocardial recovery in this subset of patients.

Meanwhile, regenerative medicine has become a concrete possibility in a constantly increasing number of clinical settings [14]. Avoiding antirejection therapy and bypassing the shortage of compatible donors, regenerative-inspired technologies mark a turning point in the future management of end-stage organ failure. Tissue decellularization to obtain cell-free extracellular matrix scaffolds able to retain the intrinsic structural integrity and biochemical properties, where the recipient’s native cells can be seeded, represents one of the directions in which regenerative medicine is progressing. Vascularized composite allografts (multiple tissues sustained by a vascular network that can be transplanted as a biological functional unit) now entail complex anatomical structures (such as skin, nerves, muscles, cartilages, etc.) that can replace the affected ones in the recipient [15]. In parallel, three-dimensional bioprinting can incorporate living cells into the fabrication process of tissue and organ analogs [16], overcoming the issues associated with donors’ availability and pursuing more personalized treatment options.

During the progression of HF, the heart gradually loses the ability to replace damaged tissues and regenerate [17]. Aiming at reactivating cardiomyocytes’ proliferation and restoring the lost myocardial tissue, regenerative medicine efforts for HF deeply differ from current therapeutic strategies that can only delay the ventricular remodeling process and simply postpone the occurrence of end-stage HF. Early attempts have focused on the direct administration of populations of stem or progenitor cells to the injured heart of animal models of HF. Although inconsistencies exist in indications, methodologies for the injections of cells, and even the type of progenitor cells used, the first reports have globally shown promising results in positively modulating the heart’s function [18,19,20,21]. The beneficial effects of these cell therapies have been attributed to several biological pathways: the mitigation of the inflammatory and immune response to the injury; the reduction in fibroblasts’ proliferation and scar formation; the prevention of cardiomyocytes’ apoptosis [18,19,20,21].

Several protocols have passed the preclinical phase successfully and have been proposed for clinical experimentation. Intracoronary infusion of cardiac progenitor cells in patients affected by single-ventricle physiology has shown the possibility of reverse remodeling, which ameliorates the ventricular contractility, volumes, and functional status of patients [22,23]. Moreover, transplantation of mesenchymal stem cells after acute myocardial infarction is currently the subject of intense investigations, including a recent randomized multicenter clinical trial [24], in order to assess their beneficial effect on the long-term development of HF after myocardial injury. Stimulating the intrinsic healing ability of the human heart and, unlike available pharmacotherapy, correcting organ dysfunction, represent the definitive aims of these studies.

Firstly, proposed in 2007 by the Giessen working group [25], reversible PAB has been reinvented as an alternative surgical option for children affected by DCM, with preserved right ventricular (RV) function. Learning from the retraining of the subpulmonary ventricle in congenitally corrected transposition of great arteries, the rationale of PAB in DCM is to promote ventricular–ventricular positive interactions and supposed molecular crosstalk [26,27], activating the repair potential of the myocardium. As a result, PAB could represent a true “regenerative surgery” for children affected by end-stage HF.

## 3. Bedside: The Clinical Efficacy of PAB

After fifteen years from the first case report of the successful application of PAB in a 2-month-old baby with DCM [25], less than 20 centers worldwide have published their clinical results [28,29,30,31]. From these findings, what we have learned is that PAB has a concrete potential to improve LV function in 30% to 80% of treated patients, when RV function is preserved. Early mortality is low (10%), comparable, or even inferior to that associated with HTX or MCS in infants [1,32,33]. No directly PAB-related deaths have been described so far. The most common causes of exitus in PAB nonresponders are progressive deterioration of LV function or lethal complications following MCS implantation, which has been implanted as a rescue strategy [28,29]. This is in keeping with the natural history of DCM, where one-year survival after diagnosis drops to 80% in high-risk profiles [34], regardless of medical and surgical therapies.

The international experience with PAB was consolidated in a multicenter study, for a total of 70 patients from 15 institutions worldwide [29]. In this cohort, PAB was adopted as part of different surgical strategies. In nine children, PAB served as a recovery strategy to wean patients with DCM following major open-heart procedures, with eight out of nine patients experiencing recovery. In the remaining 61 cases, PAB was performed as an isolated short open-chest approach. Among the latter, 8 patients underwent PAB as a bridge to HTX as an alternative to MCS, while in 53 children, PAB was intended as a true potential “regenerative surgery”. Focusing on the 61 patients who received PAB as the sole operation, outcomes are encouraging. Overall medium-term mortality is confirmed to be modest (8/61, 13%), and the rate of complete LV recovery is brilliant (34/61, 56%). In addition, 8 patients (13%) experienced a partial improvement in LV function, while 13 patients (21%) required an HTX, as a planned strategy or because of PAB nonresponders. Updates regarding these numbers are expected to further support the promising early results.

More recently, a multicenter US study enrolling 14 children banded at a median of 5 months of age highlighted some differences with respect to the German experience [30]. Functional recovery was achieved in 29% of cases (4/14 patients), while 8 patients underwent HTX and 2 expired. The authors hypothesized that the more compromised preoperative status of patients in the US cohort, as well as the substantial differences in HF etiologies (myocarditis in 40% of patients in the German registry vs. 0% in the US study), contributed to the lower observed recovery rate. In fact, PAB was attempted as a rescue strategy to prevent committing to high-risk ventricular assist device implantation [30]. In this view, it is not surprising that a supposed regenerative strategy (PAB) that relies on the residual repair potential of the native heart and organism would be less beneficial when performed “too late”. Moreover, LV noncompaction, which we found as a possible contributing factor to PAB failure in our experience [31], was unusually frequent in the US cohort (5/14 patients, 39% vs. 8/61 cases, 13% in the world network report [29]), revealing the presence of a structural detrimental substrate, traditionally associated with relevant irreversible myocardial fibrosis [35], in a consistent quote of patients.

Since 2015, we have embarked on a surgical protocol alternative to HTX to treat pediatric end-stage HF [31], including PAB. So far, we have treated with PAB a total of eight patients affected by DCM (four males, 50%) at a median age of 8 (5–10) months. In two cases (25%), the PAB strategy failed, and both patients required MCS with the Berlin Heart, followed by a successful HTX [31]. In one case (with a diagnosis of LV noncompaction and a known heterozygous mutation of the TPM1 gene and the ABCC9 gene), the PAB strategy was initially successful, allowing home discharge, but 3 months after PAB, the patient developed severe RV failure after acute pneumonia. Data regarding genetic testing in patients with DCM undergoing PAB are sparse in the scientific literature. The Giessen group never reported known mutations affecting their patients, nor gave specific recommendations on genetic testing before PAB. From the multicenter US cohort [30], only two patients were known to have genetic syndromes (including one patient with Leigh Syndrome and one patient with 12p duplication). In our center, genetic testing is always performed as part of the diagnostic protocol for patients with DCM. However, the decision to pursue the PAB strategy cannot benefit from such information, since the results of genetic tests require time, and patients usually necessitate a rapid treatment decision. The results of genetic testing are used to better guide the follow-up of patients and possibly predict the probabilities of recurrence of HF. As stated, only one patient in our cohort was found to possess a heterozygous mutation of the TPM1 gene (de novo) and the ABCC9 gene (parental).

**Figure 1:**
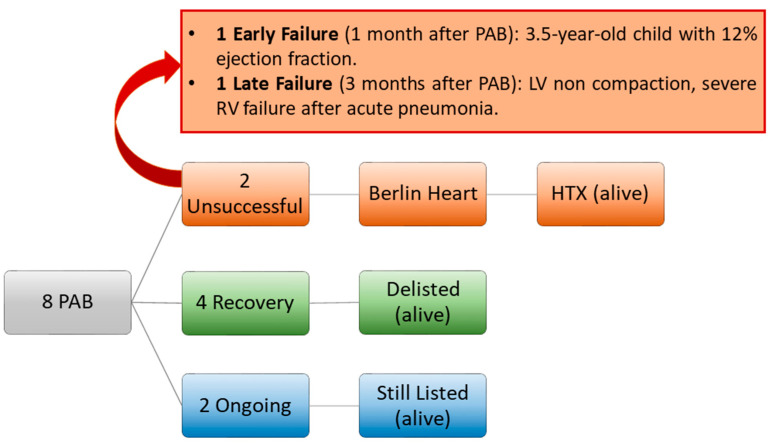
Padova experience with PAB for LV rehabilitation in DCM since 2015. HTX: heart transplantation. PAB: pulmonary artery banding. RV: right ventricle.

In four patients (50%) in our cohort, LV performance improved significantly, allowing transplantation delisting. In the remaining two patients (the most recent ones), LV function is still depressed, but clinical conditions are stable, and the patients are kept on an active transplantation waitlist (Figure 1). At a median follow-up time of 4.4 (1–5.4) years from PAB, all patients are alive and followed up regularly.

Our pre- and postoperative imaging protocol relies on transthoracic echocardiography, which is performed before surgery in the early postoperative period, at discharge, and then monthly for the first 6 months. Depending on clinical conditions, echocardiography is performed less frequently after 6 months. Paramount echocardiographic measures are RV function (fractional area change and TAPSE) and dimensions, tricuspid valve competence and Doppler inflow waves, and possible signs of pulmonary hypertension. Echocardiography is used to rule out any associated congenital heart defects, especially coronary anomalies. Left ventricular dimensions and function (ejection fraction, global circumferential, and longitudinal strain), parietal thickness, mitral valve competence and Doppler inflow waves, and intraventricular septum curvature are used to monitor the response to PAB. When feasible, we also perform cardiac magnetic resonance imaging (MRI) before PAB and 6–12 months after surgery. We suggest cardiac MRI as a standard preoperative imaging technique (when technically feasible and if hemodynamic conditions are stable) for all candidates for PAB, and a second cardiac MRI at 6–12 months would be advisable to evaluate late myocardial remodeling. Cardiac MRI is helpful to better define biventricular volumes and geometries, quantify RV and LV ejection fraction and strain parameters, rule out associated congenital heart defects and coronary anomalies, and identify and quantify myocardial fibrosis (LGE positivity), if present. This piece of information may help clinicians in the definition of the prognosis and the feasibility of myocardial remodeling after PAB.

## 4. Bedside: Caveats from Clinical Experience

### 4.1. Age

The neonatal heart is demonstrated to possess a robust capacity for myocardial tissue regeneration, through the presence of highly active cardiac progenitor cells [36]. In fact, the cardiac tissue harvested from patients with congenital heart diseases can spontaneously generate mesenchymal stem cells in vitro [37], which is indicative of a preserved endogenous capacity of myocardial repair. Unfortunately, this repair potential is age-dependent, with a progressive and marked reduction beyond one year of age [37]. There is evidence that the regeneration capacity of the adult mammalian heart is very limited, due to the incapacity of cardiomyocytes to proliferate in adulthood. However, this is not true in neonates and infants, where a residual or reversible potential for proliferation has been reported [36]. Clinical experience with infants with complex congenital heart disease (i.e., anomalous left coronary artery from the pulmonary artery) has shown that after early surgical repair (i.e., coronary reimplantation), a full myocardial recovery with normalization of LV function is possible even after myocardial ischemia [38,39]. In addition, experience with rapid two-stage arterial switch in infants affected by transposition of the great arteries, who are too old to undergo a successful arterial switch operation, has shown the possibility of LV myocardial recovery after PAB, which has been able to induce an increase in cardiac mass to allow an uneventful late repair [40,41].

While cardiomyocytes show robust replicative activity during embryonic and fetal development, with subsequent waves of proliferation [42], replication stops after birth, never to resume again, at least not significantly. Further cardiac enlargement occurs by cell hypertrophy. The withdrawal of cardiomyocytes from the cell cycle after birth profoundly impacts on the capacity of the mammalian heart to undergo repair after damage: in the mouse, loss of myocardial tissue in the fetal or early neonatal life is healed through the generation of new contractile tissue [43], while fibrosis and scarring predominate later. The reason why cardiomyocyte proliferation stops irreversibly after birth seems to be linked to biochemical and mechanical events occurring after birth, such as the increase in oxygen tension and oxidative stress [44], the lack of maternal factors [45], the changes in hormonal stimulation [46], and, most notably, pressure overload [47], as the hydrodynamic modifications occurring in the newborn circulation result in a significant increase in cardiac afterload. All these observations may suggest that the beneficial effects of PAB in young patients are related to the specific induction of acute afterload increase, with consequent cardiomyocytes proliferation and cardiac regeneration.

An ancient organ size control pathway (Hippo signaling) specifically inhibits cardiomyocyte proliferation in the adult heart [48,49]. Regulating the balance between cell differentiation, proliferation, and apoptosis, the Hippo signaling pathway also governs cardiac fibroblasts’ function and, subsequently, heart fibrosis [50], together with the endothelial response to oxidative stress, inflammation, and angiogenesis [51]. For these reasons, the inactivation of the Hippo pathway has been proposed as part of the putative strategies to promote myocardial regeneration after an injury [52]. If proven, PAB might act on these molecular pathways, modifying intracardiac pressure parameters that are detected by the cardiac mechano-sensing apparatus, which can regulate the Hippo signaling [53,54]. In fact, the dystrophin–glycoprotein complex, which links the cytoskeleton of myocardial fibers to the extracellular matrix, can regulate the Hippo pathway by binding its principal effector YAP (Yes-associated protein), modulating cardiomyocyte proliferation in mice [53]. This protein is regulated by the mechanical stress and the extracellular matrix stiffness, which are obliviously affected by the pressure overload imposed by PAB on the RV chamber.

**Figure 2:**
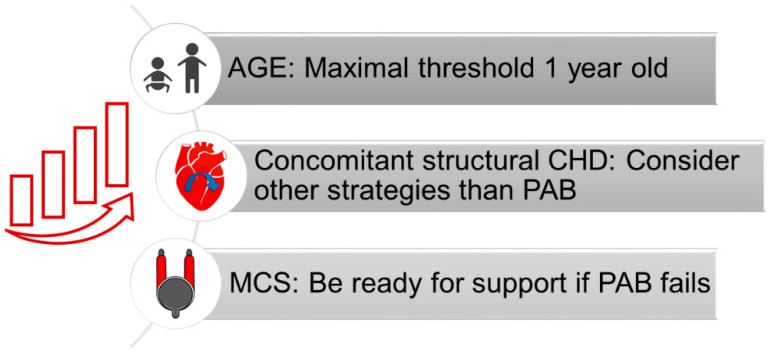
Proposed strategies to improve patients’ outcomes. CHD: congenital heart defect. MCS: mechanical support device. PAB: pulmonary artery banding.

In our experience, a possible cause of PAB failure is the age of patients [31]. Schranz et al. suggested a 6-year-old age limit for PAB in DCM [55]. However, we believe that a lower threshold should be applied. Relying on the repair ability of the young heart and the antiregenerative pathways that aging activates in the myocardium, we now consider PAB as a surgical option only in children under 1 year of age (Figure 2) [31]. A similar philosophy has emerged in the setting of retraining of the morphological LV in congenitally corrected transposition of the great arteries, where the maximal age threshold for patients to be considered for PAB has been lowered across the years. In 2005, Winlaw et al. observed that patients >16 years at PAB were unlikely to achieve a definitive anatomical repair [56]. Eight years later, Myers et al. described an increased risk of LV dysfunction when PAB was performed after 2 years of age [57]. In the most recent series, the median age at PAB is even below 1 year [58], reflecting a trend toward the anticipation of PAB in early infancy.

Although our hypotheses require further validation, surgeons should be aware that rates of PAB nonresponders in pediatric DCM and, subsequently, mortality, may be age-related. A deeper analysis of the international registries on PAB as well as targeted experimental research is hoped to clarify this topic.

### 4.2. Associated Cardiac Defects

In nine patients from the worldwide multicenter cohort [29], PAB was performed in association with other major surgical procedures (mitral valve repair/replacement, correction of an anomalous left coronary artery arising from the pulmonary artery, repair of partial anomalous pulmonary venous return, etc.). However, we consider major associated cardiac defects as relative contraindications to PAB [31], as suggested by the selection criteria proposed by Schranz et al. [55].

One of the most important advantages of PAB is the minimal surgical invasiveness, requiring neither cardioplegic arrest of the heart nor cardiopulmonary bypass. As described in adult cardiac diseases, the more profound the LV dysfunction is, the less the cardioplegic arrest of the heart is tolerated [59]. Due to the higher density of Thebesian and arterio-luminal vessels that may contribute to a more rapid washout of the cardioplegic solution [60,61], the RV is historically known to be more susceptible to ischemic injury during cardiopulmonary bypass [62,63,64,65].

On a molecular basis, in patients with HF, the ischemic/reperfusion injury associated with the cardioplegic arrest of the heart results in an exasperated inflammatory response and myocardial cytokine release when compared to nonfailing hearts [66]. Thus, the cardioplegic arrest needed to perform the surgical repair of associated intracardiac defects could impair the hypothesized immunomodulatory effects of PAB in pediatric DCM. Consequently, the abrupt overwork induced by PAB during the delicate phases of coronary reperfusion may result in acute RV failure rather than a hypertrophic positive adaptation. For this reason, we perform PAB mostly as an isolated procedure at our institution, to minimize myocardial injury and optimize the efficacy of PAB (Figure 2). In the case of substantial structural defects to be addressed, alternative strategies are considered [31].

### 4.3. MCS Backup

According to the worldwide network report by Schranz et al. [29], PAB in DCM can serve different purposes: 1. as a recovery strategy to wean patients with DCM following major open-heart procedures; 2. as a bridge to a planned HTX to avoid MCS support; 3. as a bridge to recovery. Nonresponders to PAB range from 30% to 60% of treated patients, in the European and US experiences, respectively [28,30,31]. Lower recovery rates may reflect a more compromised preoperative status, when the detrimental cascade of HF is less likely to be reverted [30]. Moreover, the immediate postoperative days represent a critical period for achieving adequate circulatory stabilization, and acute insults may dramatically result in biventricular failure [29]. In this setting, a prompt instauration of MCS is the only chance to sustain the patient until a compatible donor is available. In our experience, two children in whom PAB was unsuccessful both required temporary circulatory support with the Berlin Heart, before undergoing an uneventful HTX [31]. Similarly, seven out of eight patients who manifested an early or late deterioration after PAB in the US cohort were placed on ventricular assist device support until a compatible donor was available [30]. Moreover, in our experience, a temporary MCS has also been helpful in two more compromised patients who experienced low-cardiac-output syndrome after PAB. They were assisted on venous–arterial ECMO for a few days until a successful and uneventful weaning off.

For this reason, we strongly recommend that PAB be considered as a surgical option exclusively in patients who are eligible for long-term MCS. Complete patient assessment for MCS, family counseling, and notification to manufacturers must be integral parts of the preoperative planning of PAB (Figure 2). Since it is well-recognized that surgical volume is a determinant of MCS outcomes [67], a solid background in pediatric MCS and ECMO support is mandatory before including PAB in the institutional surgical protocol for end-stage HF. With more patients being treated with PAB, we expect further refinements of selection criteria, possibly stratifying candidates based on the risk of PAB failure, and guiding a more targeted planning of an MCS backup.

## 5. Bedside: Functional Changes Induced by PAB

The first hypotheses for the mechanisms of action of PAB have been derived from the echocardiographic follow-up of patients. PAB responders usually display a gradual improvement in LV ejection fraction, even at discharge, which becomes more evident 3 to 6 months after the operation [28,68]. Data from the worldwide network of PAB showed a brilliant recovery of LV ejection fraction in PAB responders, which increased from a mean of 20% before PAB to 44% and 60% after 3 to 6 months from the operation [29]. Concomitantly, the LV undergoes a progressive reshaping with a significant reduction in end-diastolic diameters, which decrease from a mean z-score of +7 before surgery to +4 at discharge and to normal values (+1) at the last follow-up in PAB responders [29]. The acute LV remodeling that PAB promotes also affects the severity of mitral regurgitation positively [68]. All these changes come with a drop in serum biomarkers of HF (brain-type natriuretic peptide) and a clinical improvement in patients’ functional status [29]. Our experience parallels previous findings: the four PAB responders exhibited a progressive amelioration in LV ejection fraction, which increased from a mean of 16 ± 6% before PAB, to 30 ± 8% after 3 months and 63 ± 6% at 6 months.

One of the main concerns about the efficacy of PAB is the possibility of having selected those patients affected by DCM in whom the LV would have recovered anyway, independently from PAB. Spontaneous healing is possible in 10–20% of patients with DCM, especially when a myocarditis-based mechanism is suspected [69,70]. However, functional recovery necessitates years, being extremely infrequent in the first year after the diagnosis. In fact, Wang et al. recently described a cumulative recovery rate of 1.2%, 9.3%, and 12.1% at 1, 3, and 5 years after the diagnosis, respectively, in their pediatric cohort of patients with DCM [70]. Conversely, the significant reverse remodeling and improvement in LV function that are seen just a few months after PAB support a causative role of the procedure in promoting or accelerating myocardial healing.

Novel insights into ventricular–ventricular positive interactions recruited by PAB in DCM have been provided by MRI and speckle-tracking imaging [71,72]. Comparing cardiac MRI data at baseline and one year after PAB, Latus et al. confirmed the improvements in LV dimensions and ejection fraction seen at echocardiography [71]. Surprisingly, despite the increased afterload, RV end-diastolic and end-systolic volumes remained stable, thanks to the hypertrophic adaptation. Moreover, intraventricular septal curvature displayed a significant leftward shift after PAB, which was directly correlated with the measured gradients across PAB. As novel findings, circumferential, longitudinal, and radial strain indexes improved significantly in both ventricles, together with the intraventricular septum synchrony measures [71]. These data support the primary role of the leftward shift of the intraventricular septum in mitigating LV dilatation and ameliorating LV filling/preload. Moreover, the increased intrinsic myocardial contractility revealed by strain analysis suggests a more profound effect of PAB on LV myocardial fibers, potentially due to increased recruitment and optimized working conditions.

**Figure 3:**
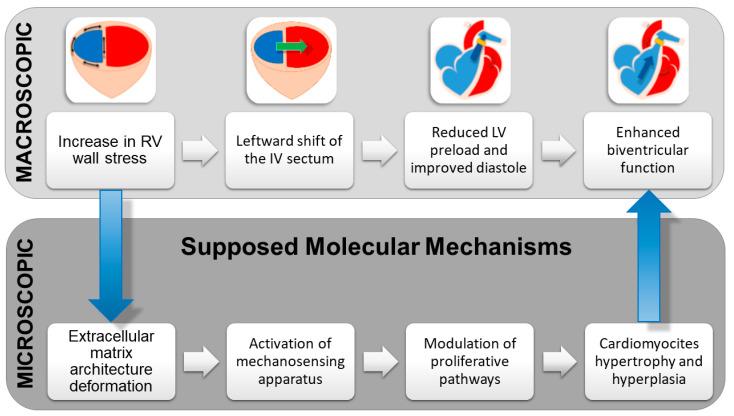
Functional changes induced by PAB and possible cellular and molecular correlates. IV: interventricular septum. LV: left ventricle. RV: right ventricle.

To summarize, PAB promotes the following hemodynamic changes (Figure 3):

- Increase in RV wall stress, which translates into RV hypertrophy and enhanced contractility due to the Anrep effect.
- Leftward shift of the interventricular septum that contributes to LV reshaping and restoration of an ellipsoidal rather than spherical shape.
- Reduction in LV preload and end-diastolic pressure, with subsequent optimization of the Frank–Starling curve and LV diastolic function.
- Decrease in LV dilatation during the whole cardiac cycle.
- Better left atrial–ventricular coupling and reduction in the severity of mitral regurgitation.
- Enhanced LV contractility.
- Reestablishment of biventricular synchrony.

## 6. Bench: A Step back to Move Forward

To date, the biological pathways recruited by PAB are still unidentified and knowledge gaps exist regarding the molecular-, cellular-, and tissue-level working mechanisms of this procedure. To test this novel therapeutic approach to HF in a controlled manner, a viable and reproducible animal model of DCM with hypothesized rescue using PAB is paramount. Yerebakan et al. proposed an innovative experimental sheep model of LV failure generated by intermittent intracoronary injections of doxorubicin treated with PAB [73]. Echocardiography 3 months after the procedure revealed better LV ejection fraction and fractional shortening, as well as smaller LV dimensions, in the treatment group. However, the authors failed to prove any difference in fibrosis or inflammatory infiltrate at terminal histopathology. Causes may be multiple. Firstly, although validated [74], doxorubicin-based HF does not reproduce the exact pathological bases of DCM. Doxorubicin causes swelling and vacuolization of cardiomyocytes, disorganization of myofibrils, and diffuse fibrosis [75], which are only partial findings in human DCM. Moreover, experimental studies focus mainly on doxorubicin’s effects on the LV, while clinical experience in cancer survivors showed that anthracycline toxicity may also affect the RV [76]. Recent findings also suggest an increased production of free radicals in the RV myocardium of doxorubicin-treated rats, revealing its possible biventricular toxicity [77]. Since preserved RV function is one of the key aspects of successful PAB application in DCM, the beneficial effects of PAB might be undermined in a doxorubicin-based model of HF. Secondly, immunohistochemistry analysis was not performed in the study by Yerebakan et al., and thus cellular modifications induced by PAB may have been underestimated. Finally, the grade of hypertrophy and hyperplasia of cardiomyocytes was not assessed. Although preliminary, we praise the efforts of these authors, since an experimental model able to elucidate the underlying mechanisms that promote ventricular–ventricular interactions and stimulate the reverse remodeling of the decompensated LV represents the cardinal setting in which to provide an evidence-based explanation of the efficacy of PAB in DCM.

PAB is hypothesized to promote the regenerative capacity of the myocardium. Cardiomyocytes can detect wall stress modifications through integrin transmembrane receptors located in the sarcolemma [78]. Moreover, the transcriptional coactivators YAP and TAZ respond to cytoskeletal deformations related to cell shape and extracellular matrix architecture [53,79]. These molecules are converging effectors of Hippo signaling [54]. Consequently, the myocardial sensing apparatus might translate the mechanical stress stimulus into the activation of one of the most important regulatory pathways of cardiomyocyte proliferation [48,49]. Experimental studies proved that the cardiac-specific deletion of the Mst kinase coactivator WW45 (a key modulator of the Hippo pathway) in mice results in cardiomegaly and increased cardiomyocyte proliferation [48]. Similar findings were observed when inducing an overexpression of YAP in the embryonic heart of mice [80]. On the contrary, the deletion of YAP impedes cardiomyocyte proliferation, causing myocardial hypoplasia and in utero death in knockout mice. Interestingly, the Hippo pathway is also involved in myocardial healing after an injury [81]. The induced cytosolic retention of YAP impedes the transcription of several antioxidant genes, with subsequent increased oxidative stress and apoptosis following ischemic/reperfusion injury in mice. Restoring the correct nuclear localization of YAP prevents mitochondrial damage, cell death, and attenuates LV dysfunction [82].

In this view, the effects of PAB could go beyond the initial Anrep effect on the RV and the optimization of the Frank–Starling curve for the LV. If demonstrated, the increased afterload caused by PAB would reactivate the proliferative potential of cardiomyocytes, stimulating a more profound LV rehabilitation. Moreover, acting as a promoter of the antioxidant defenses (through a supposed overexpression of YAP), PAB would reduce oxidative mitochondrial stress and related cell injury, which are common components of different etiologies of DCM [83].

Proteomic analysis of rats undergoing PAB demonstrated clear modifications of the energetic, antioxidant, and structural pathways induced by the increased afterload [84]. A metabolic shift from beta-oxidation to glycolysis, together with a rise in heat-shock protein expression, is seen in the RV, but also, interestingly, in the LV [85], supporting the hypothesis of molecular crosstalk between the two ventricles. In fact, high-throughput RNA sequencing proved PAB caused a significant shift in gene expression in both ventricles (involving nuclear factor κB, extracellular matrix–receptor interaction, and nucleotide oligomerization domain-like receptor signaling pathways), even in the very acute phase after PAB [86].

Peculiarly, the response to afterload augmentation between the two ventricles presents some diverging points at the protein level. Friehs et al. compared the proteomic profile of neonate rabbits undergoing aortic constriction vs. PAB, identifying increased protein expression levels for structural constituents of the myocardium, myocardial tissue development, and calcium handling only in the latter animals [87]. These findings corroborate the predominantly hypertrophic and hyperplastic responses that characterize the RV adaptation to stress from a molecular perspective as well. Thus, the identification of the microscopic and molecular correlates of macroscopic changes in biventricular geometry and function should represent the future research directions for PAB in DCM (Figure 3).

Understanding the biological working mechanisms of PAB is mandatory to achieve a wider scientific consensus on the efficacy of PAB in reverting HF, with a consequent higher adoption of this procedure as part of treatment paradigms for pediatric HF. Children with DCM will benefit from a simple and less invasive surgical option that allows the preservation of the native heart, postponing, or, hopefully, avoiding the need for HTX and MCS. Moreover, molecular pathways promoted by PAB could serve as targets for actionable and novel treatment concepts. Therapeutically promising up- or down-regulated molecules identified in this particular population could be modulated by known pharmacologic inhibitors or activators to further optimize the clinical results of PAB in DCM and, eventually, bypass surgical treatment.

### Limitations

To date, one of most limiting factors that contributes to the skepticism towards the use of PAB in DCM is the still small number of treated patients. Our cohort, although it represents one of the largest reported single-center experiences with this technique, relies on a relatively small number of patients, which limits the translatability of our findings. We hope that our work would stimulate the prospective enrollment of a greater number of patients to be considered for this treatment.

Furthermore, the possible biological working mechanisms of PAB that we hypothesized in the present work still require validation, which we aimed to encourage in future experimental research.

## 7. Conclusion: Stalemate or Turning Point?

In clinical practice, PAB proved to have a concrete potential in restoring LV function in one out of two children affected by end-stage DCM. Through the re-establishment of adequate LV preload, ellipsoidal shape, and biventricular synchrony, PAB enhances myocardial contractility and promotes ventricular–ventricular positive interactions in selected patients. However, several surgical centers are still reluctant to adopt this technique, because the precise biological pathways recruited by PAB and the ultimate clinical outcomes remain unknown. To date, we can propose only speculative hypotheses derived from ongoing animal experimentations on myocardial healing after an injury and from human data in similar clinical settings, such as the morphological LV retraining in congenitally corrected transposition of the great arteries. Understanding the cellular and molecular working mechanisms of PAB is mandatory to provide an evidence-based explanation in support of this approach. Experimental and targeted research is awaited to extend the implementation of PAB into the surgical toolbox for the treatment of pediatric HF and refine its efficacy.

## Data Availability

Not applicable.

## References

[1] Rossano J.W., Cherikh W.S., Chambers D.C., Goldfarb S., Hayes D.J., Khush K.K., Kucheryavaya A.Y., Toll A.E., Levvey B.J., Meiser B. (2018). The International Thoracic Organ Transplant Registry of the International Society for Heart and Lung Transplantation: Twenty-first pediatric heart transplantation report-2018; Focus theme: Multiorgan Transplantation. J. Heart Lung Transplant..

[2] Shaddy R.E., George A.T., Jaecklin T., Lochlainn E.N., Thakur L., Agrawal R., Solar-Yohay S., Chen F., Rossano J.W., Severin T. (2018). Systematic Literature Review on the Incidence and Prevalence of Heart Failure in Children and Adolescents. Pediatr. Cardiol..

[3] Schultheiss H.-P., Fairweather D., Caforio A.L.P., Escher F., Hershberger R.E., Lipshultz S.E., Liu P.P., Akira M., Mazzanti A., McMurray J. (2019). Dilated cardiomyopathy. Nat. Rev. Dis. Primers.

[4] Weintraub R.G., Semsarian C., Macdonald P. (2017). Dilated cardiomyopathy. Lancet.

[5] Kirk R., Dipchand A.I., Davies R.R., Miera O., Chapman G., Conway J., Denfield S., Gossett J.G., Johnson J., McCulloch M. (2020). ISHLT consensus statement on donor organ acceptability and management in pediatric heart transplantation. J. Heart Lung Transplant..

[6] de By T.M.M.H., Antonides C.F.J., Schweiger M., Sliwka J., Davies B., Berger F., Hubler M., Ozbaran M., Maruszewski B., Napoleone C.P. (2020). The European Registry for Patients with Mechanical Circulatory Support (EUROMACS): Second EUROMACS Paediatric (Paedi-EUROMACS) report. Eur. J. Cardio-Thorac. Surg..

[7] Lorts A., Conway J., Schweiger M., Adachi I., Amdani S., Auerbach S.R., Barr C., Bleiweis M.S., Blume E.D., Burstein D.S. (2021). ISHLT consensus statement for the selection and management of pediatric and congenital heart disease patients on ventricular assist devices Endorsed by the American Heart Association. J. Heart Lung Transplant..

[8] Thangappan K., Zafar F., Lorts A., Adachi I., Rosenthal D., Rossano J., Maeda K., Morales D.L. (2022). MILESTONE: More Than 1200 Children Bridged to Heart Transplantation with Mechanical Circulatory Support. ASAIO J..

[9] Chen J.M., Canter C.E., Hsu D.T., Kindel S.J., Law Y.M., McKeever J.E., Pahl E., Schumacher K.R. (2018). Current Topics and Controversies in Pediatric Heart Transplantation: Proceedings of the Pediatric Heart Transplantation Summit 2017. World J. Pediatr. Congenit. Heart Surg..

[10] Zangwill S. (2017). Five decades of pediatric heart transplantation: Challenges overcome, challenges remaining. Curr. Opin. Cardiol..

[11] Zafar F., Conway J., Bleiweis M.S., Al-Aklabi M., Ameduri R., Barnes A., Bearl D.W., Buchholz H., Church S., Do N.L. (2021). Berlin Heart EXCOR and ACTION post-approval surveillance study report. J. Heart Lung Transplant..

[12] Chaggar P.S., Williams S.G., Yonan N., Fildes J., Venkateswaran R., Shaw S.M. (2016). Myocardial recovery with mechanical circulatory support. Eur. J. Heart Fail..

[13] Delmo E.M.J., Javier M.F.D.M., Böthig D., Rüffer A., Cesnjevar R., Dandel M., Hetzer R. (2021). Heart failure in the young: Insights into myocardial recovery with ventricular assist device support. Cardiovasc. Diagn. Ther..

[14] Edgar L., Pu T., Porter B., Aziz J.M., La Pointe C., Asthana A., Orlando G. (2020). Regenerative medicine, organ bioengineering and transplantation. Br. J. Surg..

[15] Adil A., Xu M., Haykal S. (2022). Recellularization of Bioengineered Scaffolds for Vascular Composite Allotransplantation. Front. Surg..

[16] Pourmasoumi P., Moghaddam A., Nemati Mahand S., Heidari F., Salehi Moghaddam Z., Arjmand M., Kühnert I., Kruppke B., Wiesmann H.-P., Khonakdar H.A. (2022). A review on the recent progress, opportunities, and challenges of 4D printing and bioprinting in regenerative medicine. J. Biomater. Sci. Polym. Ed..

[17] Ko T., Nomura S. (2022). Manipulating Cardiomyocyte Plasticity for Heart Regeneration. Front. Cell Dev. Biol..

[18] Mills W.R., Mal N., Kiedrowski M.J., Unger R., Forudi F., Popovic Z.B., Penn M.S., Laurita K.R. (2007). Stem cell therapy enhances electrical viability in myocardial infarction. J. Mol. Cell. Cardiol..

[19] Müller-Ehmsen J., Krausgrill B., Burst V., Schenk K., Neisen U.C., Fries J.W.U., Fleischmann B.K., Hescheler J., Schwinger R.H.G. (2006). Effective engraftment but poor mid-term persistence of mononuclear and mesenchymal bone marrow cells in acute and chronic rat myocardial infarction. J. Mol. Cell. Cardiol..

[20] Liu Y., Guo J., Zhang P., Zhang S., Chen P., Ma K., Zhou C. (2004). Bone marrow mononuclear cell transplantation into heart elevates the expression of angiogenic factors. Microvasc. Res..

[21] Kim Y.-H. (2003). Intramyocardial transplantation of circulating CD34+ cells: Source of stem cells for myocardial regeneration. J. Korean Med. Sci..

[22] Ishigami S., Ohtsuki S., Eitoku T., Ousaka D., Kondo M., Kurita Y., Hirai K., Fukushima Y., Baba K., Goto T. (2017). Intracoronary Cardiac Progenitor Cells in Single Ventricle Physiology: The PERSEUS (Cardiac Progenitor Cell Infusion to Treat Univentricular Heart Disease) Randomized Phase 2 Trial. Circ. Res..

[23] Makkar R.R., Kereiakes D.J., Aguirre F., Kowalchuk G., Chakravarty T., Malliaras K., Francis G.S., Povsic T.J., Schatz R., Traverse J.H. (2020). Intracoronary ALLogeneic heart STem cells to Achieve myocardial Regeneration (ALLSTAR): A randomized, placebo-controlled, double-blinded trial. Eur. Heart J..

[24] Attar A., Monabati A., Montaseri M., Vosough M., Hosseini S.A., Kojouri J., Abdi-Ardekani A., Izadpanah P., Azarpira N., Pouladfar G. (2022). Transplantation of mesenchymal stem cells for prevention of acute myocardial infarction induced heart failure: Study protocol of a phase III randomized clinical trial (Prevent-TAHA8). Trials.

[25] Schranz D., Veldman A., Bartram U., Michel-Behnke I., Bauer J., Akintürk H. (2007). Pulmonary artery banding for idiopathic dilative cardiomyopathy: A novel therapeutic strategy using an old surgical procedure. J. Thorac. Cardiovasc. Surg..

[26] Koestenberger M., Sallmon H., Avian A., Cantinotti M., Gamillscheg A., Kurath-Koller S., Schweintzger S., Hansmann G. (2019). Ventricular-ventricular interaction variables correlate with surrogate variables of clinical outcome in children with pulmonary hypertension. Pulm. Circ..

[27] Friedberg M.K. (2018). Imaging Right-Left Ventricular Interactions. JACC Cardiovasc. Imaging.

[28] Schranz D., Rupp S., Müller M., Schmidt D., Bauer A., Valeske K., Michel-Behnke I., Jux C., Apitz C., Thul J. (2013). Pulmonary artery banding in infants and young children with left ventricular dilated cardiomyopathy: A novel therapeutic strategy before heart transplantation. J. Heart Lung Transplant..

[29] Schranz D., Akintuerk H., Bailey L. (2018). Pulmonary Artery Banding for Functional Regeneration of End-Stage Dilated Cardiomyopathy in Young Children: World Network Report. Circulation.

[30] Spigel Z.A., Razzouk A., Nigro J.J., Karamlou T.B., Kavarana M.N., Roeser M.E., Adachi I. (2020). Pulmonary Artery Banding for Children with Dilated Cardiomyopathy: US Experience. Semin. Thorac. Cardiovasc. Surg. Pediatr. Card. Surg. Annu..

[31] Ponzoni M., Frigo A.C., Castaldi B., Cerutti A., Di Salvo G., Vida V.L., Padalino M.A. (2021). Surgical strategies for the management of end-stage heart failure in infants and children: A 15-year experience with a patient-tailored approach. Artif. Organs.

[32] Morales D.L.S., Adachi I., Peng D.M., Sinha P., Lorts A., Fields K., Conway J., Louis J.D.S., Cantor R., Koehl D. (2020). Fourth Annual Pediatric Interagency Registry for Mechanical Circulatory Support (Pedimacs) Report. Ann. Thorac. Surg..

[33] Rossano J.W., Singh T.P., Cherikh W.S., Chambers D.C., Harhay M.O., Hayes D.J., Hsich E., Khush K.K., Meiser B., Potena L. (2019). The International Thoracic Organ Transplant Registry of the International Society for Heart and Lung Transplantation: Twenty-second pediatric heart transplantation report—2019; Focus theme: Donor and recipient size match. J. Heart Lung Transplant..

[34] Rossano J.W., Kantor P.F., Shaddy R.E., Shi L., Wilkinson J.D., Jefferies J.L., Czachor J.D., Razoky H., Wirtz H.S., Depre C. (2020). Elevated Heart Rate and Survival in Children with Dilated Cardiomyopathy: A Multicenter Study from the Pediatric Cardiomyopathy Registry. J. Am. Heart Assoc..

[35] Nucifora G., Aquaro G.D., Pingitore A., Masci P.G., Lombardi M. (2011). Myocardial fibrosis in isolated left ventricular non-compaction and its relation to disease severity. Eur. J. Heart Fail..

[36] Sharma S., Mishra R., Bigham G.E., Wehman B., Khan M.M., Xu H., Saha P., Goo Y.A., Datla S.R., Chen L. (2017). A Deep Proteome Analysis Identifies the Complete Secretome as the Functional Unit of Human Cardiac Progenitor Cells. Circ. Res..

[37] Traister A., Patel R., Huang A., Patel S., Plakhotnik J., Lee J.E., Medina M.G., Welsh C., Ruparel P., Zhang L. (2018). Cardiac regenerative capacity is age- and disease-dependent in childhood heart disease. PLoS ONE.

[38] Latus H., Gummel K., Rupp S., Mueller M., Jux C., Kerst G., Akintuerk H., Bauer J., Schranz D., Apitz C. (2014). Cardiovascular magnetic resonance assessment of ventricular function and myocardial scarring before and early after repair of anomalous left coronary artery from the pulmonary artery. J. Cardiovasc. Magn. Reson..

[39] Haubner B.J., Schneider J., Schweigmann U., Schuetz T., Dichtl W., Velik-Salchner C., Stein J.-I., Penninger J.M. (2016). Functional Recovery of a Human Neonatal Heart after Severe Myocardial Infarction. Circ. Res..

[40] Duan Y., Sun Y., Dong S., Du C., Yan J. (2022). Two-Stage Arterial Switch for Transposition of the Great Vessels in Older Children. Ann. Thorac. Surg..

[41] Boutin C., Jonas R.A., Sanders S.P., Wernovsky G., Mone S.M., Colan S.D. (1994). Rapid two-stage arterial switch operation. Acquisition of left ventricular mass after pulmonary artery banding in infants with transposition of the great arteries. Circulation.

[42] Sedmera D., Reckova M., DeAlmeida A., Coppen S.R., Kubalak S.W., Gourdie R.G., Thompson R.P. (2003). Spatiotemporal pattern of commitment to slowed proliferation in the embryonic mouse heart indicates progressive differentiation of the cardiac conduction system. Anat. Rec. Part A Discov. Mol. Cell. Evol. Biol..

[43] Porrello E.R., Mahmoud A.I., Simpson E., Hill J.A., Richardson J.A., Olson E.N., Sadek H.A. (2011). Transient regenerative potential of the neonatal mouse heart. Science.

[44] Puente B.N., Kimura W., Muralidhar S.A., Moon J., Amatruda J.F., Phelps K.L., Grinsfelder D., Rothermel B.A., Chen R., Garcia J.A. (2014). The oxygen-rich postnatal environment induces cardiomyocyte cell-cycle arrest through DNA damage response. Cell.

[45] Zacchigna S., Martinelli V., Moimas S., Colliva A., Anzini M., Nordio A., Costa A., Rehman M., Vodret S., Pierro C. (2018). Paracrine effect of regulatory T cells promotes cardiomyocyte proliferation during pregnancy and after myocardial infarction. Nat. Commun..

[46] Hirose K., Payumo A.Y., Cutie S., Hoang A., Zhang H., Guyot R., Lunn D., Bigley R.B., Yu H., Wang J. (2019). Evidence for hormonal control of heart regenerative capacity during endothermy acquisition. Science.

[47] Canseco D.C., Kimura W., Garg S., Mukherjee S., Bhattacharya S., Abdisalaam S., Das S., Asaithamby A., Mammen P.P., Sadek H.A. (2015). Human ventricular unloading induces cardiomyocyte proliferation. J. Am. Coll. Cardiol..

[48] Heallen T., Zhang M., Wang J., Bonilla-Claudio M., Klysik E., Johnson R.L., Martin J.F. (2011). Hippo pathway inhibits Wnt signaling to restrain cardiomyocyte proliferation and heart size. Science.

[49] Heallen T., Morikawa Y., Leach J., Tao G., Willerson J.T., Johnson R.L., Martin J.F. (2013). Hippo signaling impedes adult heart regeneration. Development.

[50] Tsai C.-R., Martin J.F. (2022). Hippo signaling in cardiac fibroblasts during development, tissue repair, and fibrosis. Curr. Top. Dev. Biol..

[51] Zhang W., Li Q.-Q., Gao H.-Y., Wang Y.-C., Cheng M., Wang Y.-X. (2022). The regulation of yes-associated protein/transcriptional coactivator with PDZ-binding motif and their roles in vascular endothelium. Front. Cardiovasc. Med..

[52] Valizadeh A., Asghari S., Mansouri P., Alemi F., Majidinia M., Mahmoodpoor A., Yousefi B. (2022). The Roles of Signaling Pathways in Cardiac Regeneration. Curr. Med. Chem..

[53] Morikawa Y., Heallen T., Leach J., Xiao Y., Martin J.F. (2017). Dystrophin-glycoprotein complex sequesters Yap to inhibit cardiomyocyte proliferation. Nature.

[54] Panciera T., Azzolin L., Cordenonsi M., Piccolo S. (2017). Mechanobiology of YAP and TAZ in physiology and disease. Nat. Rev. Mol. Cell Biol..

[55] Schranz D., Recla S., Malcic I., Kerst G., Mini N., Akintuerk H. (2019). Pulmonary artery banding in dilative cardiomyopathy of young children: Review and protocol based on the current knowledge. Transl. Pediatr..

[56] Winlaw D.S., McGuirk S.P., Balmer C., Langley S.M., Griselli M., Stümper O., De Giovanni J.V., Wright J.G., Thorne S., Barron D.J. (2005). Intention-to-treat analysis of pulmonary artery banding in conditions with a morphological right ventricle in the systemic circulation with a view to anatomic biventricular repair. Circulation.

[57] Myers P.O., del Nido P.J., Geva T., Bautista-Hernandez V., Chen P., Mayer J.E.J., Emani S.M. (2013). Impact of age and duration of banding on left ventricular preparation before anatomic repair for congenitally corrected transposition of the great arteries. Ann. Thorac. Surg..

[58] Ibrahimiye A.N., Mainwaring R.D., Patrick W.L., Downey L., Yarlagadda V., Hanley F.L. (2017). Left Ventricular Retraining and Double Switch in Patients with Congenitally Corrected Transposition of the Great Arteries. World J. Pediatr. Congenit. Heart Surg..

[59] Trescher K., Bauer M., Dietl W., Hallström S., Wick N., Wolfsberger M., Ullrich R., Jurgens G., Wolner E., Podesser B.K. (2009). Improved myocardial protection in the failing heart by selective endothelin-A receptor blockade. J. Thorac. Cardiovasc. Surg..

[60] Ansari A. (2001). Anatomy and clinical significance of ventricular Thebesian veins. Clin. Anat..

[61] Crystal G.J., Pagel P.S. (2018). Right Ventricular Perfusion: Physiology and Clinical Implications. Anesthesiology.

[62] Mankad P.S., Yacoub M.H. (1993). Systolic and diastolic function of both ventricles after prolonged cardioplegic arrest. Ann. Thorac. Surg..

[63] Abdul-Ghani S., Skeffington K.L., Kim M., Moscarelli M., Lewis P.A., Heesom K., Fiorentino F., Emanueli C., Reeves B.C., Punjabi P.P. (2022). Effect of cardioplegic arrest and reperfusion on left and right ventricular proteome/phosphoproteome in patients undergoing surgery for coronary or aortic valve disease. Int. J. Mol. Med..

[64] Donauer M., Schneider J., Jander N., Beyersdorf F., Keyl C. (2020). Perioperative Changes of Right Ventricular Function in Cardiac Surgical Patients Assessed by Myocardial Deformation Analysis and 3-Dimensional Echocardiography. J. Cardiothorac. Vasc. Anesth..

[65] Murphy C.O., Pan-Chih Gott J.P., Guyton R.A. (1995). Microvascular reactivity after crystalloid, cold blood, and warm blood cardioplegic arrest. Ann. Thorac. Surg..

[66] Kortekaas K.A., Lindeman J.H., Versteegh M.I., van Beelen E., Kleemann R., Klautz R.J. (2013). Heart failure determines the myocardial inflammatory response to injury. Eur. J. Heart Fail..

[67] Morales D.L.S., Rossano J.W., VanderPluym C., Lorts A., Cantor R., St. Louis J.D., Koeh D., Sutcliffe D.L., Adachi I., Kirklin J.K. (2019). Third Annual Pediatric Interagency Registry for Mechanical Circulatory Support (Pedimacs) Report: Preimplant Characteristics and Outcomes. Ann. Thorac. Surg..

[68] Di Candia A., Castaldi B., Bordin G., Cerutti A., Reffo E., Biffanti R., Di Salvo G., Vida V.L., Padalino M. (2020). A Pulmonary Artery Banding for Ventricular Rehabilitation in Infants with Dilated Cardiomyopathy: Early Results in a Single-Center Experience. Front. Pediatr..

[69] Kim D.-H., Choi E.S., Kwon B.S., Park C.S., Cha S.G., Baek J.S., Yu J.J., Kim Y.-H., Yun T.-J. (2021). Development of Cardiac Events and Functional Recovery Prediction Models for Pediatric Dilated Cardiomyopathy. Front. Pediatr..

[70] Wang P.-Y., Tseng W.-C., Fu C.-M., Wu M.-H., Wang J.-K., Chen Y.-S., Chou N.-K., Wang S.-S., Chiu S.-N., Lin M.-T. (2021). Long-Term Outcomes and Prognosticators of Pediatric Primary Dilated Cardiomyopathy in an Asian Cohort. Front. Pediatr..

[71] Latus H., Hachmann P., Gummel K., Recla S., Voges I., Mueller M., Bauer J., Yerebakan C., Akintuerk H., Apitz C. (2016). Biventricular response to pulmonary artery banding in children with dilated cardiomyopathy. J. Heart Lung Transplant..

[72] Liu Y.-H., Chen Y.-S., Lin M.-T., Chen C.-A. (2019). Improved Left Ventricular Strain and Dyssynchrony after Pulmonary Artery Banding in an Infant with End-Stage Dilated Cardiomyopathy: Insights from Three-Dimensional Speckle Tracking. Pediatr. Cardiol..

[73] Yerebakan C., Boltze J., Elmontaser H., Yoruker U., Latus H., Khalil M., Ostermayer S., Steinbrenner B., Apitz C., Schneider M. (2019). Effects of pulmonary artery banding in doxorubicin-induced left ventricular cardiomyopathy. J. Thorac. Cardiovasc. Surg..

[74] Borenstein N., Bruneval P., Behr L., Laborde F., Montarras D., Daurès J.P., Derumeaux G., Pouchelon J.-L., Chetboul V. (2006). An ovine model of chronic heart failure: Echocardiographic and tissue Doppler imaging characterization. J. Card. Surg..

[75] O’Connell J.L., Romano M.M.D., Campos Pulici E.C., Carvalho E.E.V., de Souza F.R., Tanaka D.M., Maciel B.C., Salgado H.C., Fazan R., Rossi M.A. (2017). Short-term and long-term models of doxorubicin-induced cardiomyopathy in rats: A comparison of functional and histopathological changes. Exp. Toxicol. Pathol..

[76] Boczar K.E., Aseyev O., Sulpher J., Johnson C., Burwash I.G., Turek M., Dent S., Dwivedi G. (2016). Right heart function deteriorates in breast cancer patients undergoing anthracycline-based chemotherapy. Echo Res. Pract..

[77] Rahmanifard M., Vessal M., Noorafshan A., Karbalay-Doust S., Naseh M. (2021). The Protective Effects of Coenzyme Q10 and Lisinopril against Doxorubicin-Induced Cardiotoxicity in Rats: A Stereological and Electrocardiogram Study. Cardiovasc. Toxicol..

[78] Yu H.-L., Hwang S.-P.L. (2022). Zebrafish integrin a3b is required for cardiac contractility and cardiomyocyte proliferation. Biochem. Biophys. Res. Commun..

[79] Chakraborty S., Njah K., Pobbati A.V., Lim Y.B., Raju A., Lakshmanan M., Tergaonkar V., Lim C.T., Hong W. (2017). Agrin as a Mechanotransduction Signal Regulating YAP through the Hippo Pathway. Cell Rep..

[80] Zhou J. (2014). An emerging role for Hippo-YAP signaling in cardiovascular development. J. Biomed. Res..

[81] Xin M., Kim Y., Sutherland L.B., Murakami M., Qi X., McAnally J., Porrello E.R., Mahmoud A.I., Tan W., Shelton J.M. (2013). Hippo pathway effector Yap promotes cardiac regeneration. Proc. Natl. Acad. Sci. USA.

[82] Ambrosini S., Montecucco F., Kolijn D., Pedicino D., Akhmedov A., Mohammed S.A., Herwig M., Gorica E., Szabó P.L., Weber L. (2022). Methylation of the Hippo effector YAP by the methyltransferase SETD7 drives myocardial ischemic injury: A translational study. Cardiovasc. Res..

[83] Spyropoulos F., Sorrentino A., van der Reest J., Yang P., Waldeck-Weiermair M., Steinhorn B., Eroglu E., Saeedi Saravi S.S., Yu P., Haigis M. (2022). Metabolomic and transcriptomic signatures of chemogenetic heart failure. Am. J. Physiol. Heart Circ. Physiol..

[84] Faber M.J., Dalinghaus M., Lankhuizen I.M., Bezstarosti K., Dekkers D.H.W., Duncker D.J., Helbing W.A., Lamers J.M.J. (2005). Proteomic changes in the pressure overloaded right ventricle after 6 weeks in young rats: Correlations with the degree of hypertrophy. Proteomics.

[85] Faber M.J., Dalinghaus M., Lankhuizen I.M., Bezstarosti K., Verhoeven A.J.M., Duncker D.J., Helbing W.A., Lamers J.M.J. (2007). Time dependent changes in cytoplasmic proteins of the right ventricle during prolonged pressure overload. J. Mol. Cell. Cardiol..

[86] Cao Y., Li Y., Wu M., Song J., Zhang M., Duan Y., Jiang K., Zhou X., Zhang Y. (2020). RNA-sequencing analysis of gene expression in a rat model of acute right heart failure. Pulm. Circ..

[87] Friehs I., Cowan D.B., Choi Y.-H., Black K.M., Barnett R., Bhasin M.K., Daly C., Dillon S.J., Libermann T.A., McGowan F.X. (2013). Pressure-overload hypertrophy of the developing heart reveals activation of divergent gene and protein pathways in the left and right ventricular myocardium. Am. J. Physiol. Heart Circ. Physiol..

